# A systematic review of factors influencing student ratings in undergraduate medical education course evaluations

**DOI:** 10.1186/s12909-015-0311-8

**Published:** 2015-03-05

**Authors:** Sarah Schiekirka, Tobias Raupach

**Affiliations:** 1 Department of Cardiology and Pneumology, University Hospital Göttingen, Göttingen, Germany; 2 Study Deanery of Göttingen Medical School, Göttingen, Germany; 3 Department of Clinical, Educational and Health Psychology, University College London, London, UK

**Keywords:** Undergraduate medical education, Evaluation, Student rating, Validity, Influence, Bias

## Abstract

**Background:**

Student ratings are a popular source of course evaluations in undergraduate medical education. Data on the reliability and validity of such ratings have mostly been derived from studies unrelated to medical education. Since medical education differs considerably from other higher education settings, an analysis of factors influencing overall student ratings with a specific focus on medical education was needed.

**Methods:**

For the purpose of this systematic review, online databases (PubMed, PsycInfo and Web of Science) were searched up to August 1st, 2013. Original research articles on the use of student ratings in course evaluations in undergraduate medical education were eligible for inclusion. Included studies considered the format of evaluation tools and assessed the association of independent and dependent (i.e., overall course ratings) variables. Inclusion and exclusion criteria were checked by two independent reviewers, and results were synthesised in a narrative review.

**Results:**

Twenty-five studies met the inclusion criteria. Qualitative research (2 studies) indicated that overall course ratings are mainly influenced by student satisfaction with teaching and exam difficulty rather than objective determinants of high quality teaching. Quantitative research (23 studies) yielded various influencing factors related to four categories: student characteristics, exposure to teaching, satisfaction with examinations and the evaluation process itself. Female gender, greater initial interest in course content, higher exam scores and higher satisfaction with exams were associated with more positive overall course ratings.

**Conclusions:**

Due to the heterogeneity and methodological limitations of included studies, results must be interpreted with caution. Medical educators need to be aware of various influences on student ratings when developing data collection instruments and interpreting evaluation results. More research into the reliability and validity of overall course ratings as typically used in the evaluation of undergraduate medical education is warranted.

**Electronic supplementary material:**

The online version of this article (doi:10.1186/s12909-015-0311-8) contains supplementary material, which is available to authorized users.

## Background

Student ratings are a popular data source of course evaluations in higher education, and a number of studies have assessed their reliability and validity as well as factors potentially impacting on evaluation results. There are four dimensions of teaching quality (structural and procedural aspects of teaching, learning outcome and individual teacher performance [1]), but hardly any evaluation tool covers all four of them. More importantly, most studies on evaluation do not state explicitly the dimension(s) to which they are referring. As a consequence, evaluation data based on (overall) student ratings may represent any – and in fact more than one – of the four dimensions without this being apparent to teaching coordinators. However, clarity about the construct underlying evaluation data is a prerequisite for the validity of evaluation data as well as the fairness of decisions derived from them.

The majority of studies on evaluation were not done in medical schools but different higher education settings. Recommendations derived from studies in non-medical settings [2] cannot be directly applied to undergraduate medical education. As early as 1986, Scott et al. noted considerable differences when comparing their findings (obtained from a sample of medical students) to those of earlier studies unrelated to medical education [3]. Such differences are conceivable given that undergraduate medical curricula differ from other higher education curricula in many respects (for review see [4]). For example, clinical teaching is a unique feature of medical education, and problem-based learning is used less extensively in other higher education curricula. Compared to other higher education curricula, undergraduate medical education provides students with less choice regarding their courses and teachers [5]; at the same time, teaching within in a course is usually delivered by a number of different teachers. Thus, differences between medical and non-medical education relate to the structure of the curriculum, the way courses are run, specific teaching formats and student-teacher relationships [6]. Finally, the continued preferred use of multiple choice questions in many medical schools further distinguishes medical education from other subjects. This is an important difference given that the perceived difficulty and fairness of examinations impacts on student satisfaction with courses [7]. The differences between medical education and other higher education curricula pertain to all four dimensions of teaching quality: structure (fewer choice options), processes (teaching formats), learning outcome (e.g., competencies) and individual teachers (multiple roles in teaching and patient care), thus necessitating a critical appraisal of the use of student ratings with a specific focus on medical education.

The aim of this systematic review was to answer the following research question: What factors influence student ratings in undergraduate medical education course evaluations?

We hypothesised that we would be able to identify specific factors that need to be taken into account when designing evaluation instruments for undergraduate medical education courses. This study did not aim to provide an overview of available evaluation tools (including their psychometric properties) as this issue has been addressed in another recent review [8]. Unlike another recent publication [2], this review did not focus on ‘teaching effectiveness’ as overall course ratings do not necessarily measure effectiveness (they might do, but unless the underlying construct is well defined and transparent to both teachers and learners, this cannot be taken for granted). The term ‘effectiveness’ may be related to individual teachers’ performance or student learning outcome. Thus, evaluations aimed at targeting teaching effectiveness might relate to two of the four dimensions of high-quality teaching. However, in the absence of a universal definition of ‘effectiveness’ and without being explicit about what exactly is being measured by overall student ratings, these data cannot be assumed to reflect a comprehensive representation of either teacher performance or learning outcome. In fact, a thorough definition of the construct underlying ‘high quality teaching’ is needed to create an evaluation tool and derive meaningful interpretations from the data obtained. Otherwise, evaluation results are subject to confounding by factors unrelated to the construct itself. Importantly, the same influencing factor may be a confounder or a valid contributor, depending on the underlying construct of ‘high quality teaching’. This is why the term ‘influencing factor’ (instead of confounding factor) is used throughout this manuscript.

## Methods

### Search strategy

Online databases (PubMed, PsycInfo and Web of Science) were searched up to August 1st, 2013 with the terms: ‘medical education’, ‘medical school’, ‘medical curriculum’, ‘medical curricula’, ‘teaching’, ‘evaluation’, ‘evaluation methods’, ‘evaluation instruments’, ‘course evaluation’, ‘program evaluation’, ‘student’, ‘student ratings’, ‘reliability’, ‘validity’, and combinations of these. A hand-search of the reference lists of included studies was also carried out, and leading researchers in the field were contacted. Studies identified by these searches were screened for eligibility by both authors with 96.2% agreement. Details on the year(s) of study, the country/countries in which studies were conducted, study design, student samples, evaluation tools used and main results of each study were extracted and compiled into a table independently by both authors. The data abstraction form was derived from a tool used for a previous review [9] and aligned to the aims of the present study by the authors. All discrepancies were checked against the study papers, discussed and resolved. This was not a registered review, and we did not use a pre-specified review-protocol. However, we adhered to the principles for the preparation of systematic reviews [10]. Please refer to the Additional file 1 (PRISMA Checklist) for further details.

### Inclusion and exclusion criteria

We included primary and secondary analyses of prospective randomised controlled trials, observational quantitative studies and qualitative research. We only included original articles written in English and published in peer-reviewed journals. Studies were included if they considered the format of an evaluation tool, clearly defined dependent (overall student ratings) and independent variables and reported how they were related to each other. We focused on student overall ratings of undergraduate medical education (as opposed to individual teachers). We considered articles using one single overall rating as well as articles using mean scores of a series of ratings of different questions related to teaching quality (see Additional file 2: Table S1). Review articles, personal communications to editors, commentaries, editorials, and studies on resource allocation based on evaluation results were excluded. We also excluded studies unrelated to undergraduate medical education (e.g., reports on evaluation of continuing medical education activities or postgraduate education), studies on individual teacher evaluation and publications merely reporting results of the application of evaluation instruments but not assessing the instruments themselves.

### Data analysis and presentation

The quality of quantitative studies was assessed using the medical education research study quality instrument (MERSQI) that consists of ten items reflecting six domains of study quality (study design, sampling, type of data, validity, analysis, and outcomes) [11]. Due to considerable heterogeneity between included studies and the wide variety of interventions and outcome measures used, results could not be pooled statistically. Consequently, the data extracted from included studies are reported in a table, and the evidence is synthesized in a narrative review. This was a systematic review not involving any original patient or subject data. Thus, ethics approval for this study was not required.

## Results

### Search results

**Figure 1:**
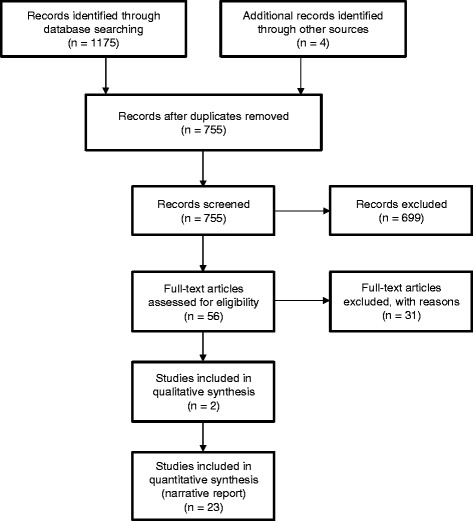
**PRISMA flowchart of the study selection and exclusion process.**

The study selection and exclusion process is outlined in Figure 1. The electronic literature search yielded 751 articles, and four potentially relevant publications were identified in reference lists of full text articles. For 56 of the 755 publications, eligibility could not be determined from the abstract so full text versions were retrieved and studied in detail. A total of 730 studies were excluded, mainly due to them not reporting original research or describing the results of applications of evaluation instruments without assessing reliability or validity of the instruments themselves (n = 623). A smaller proportion of excluded studies was unrelated to undergraduate medical education (n = 72), and even fewer focused on teacher evaluation rather than course evaluation (n = 34). One study discussing resource allocation on the basis of evaluation results was excluded as it did not comment on the reliability and validity or factors potentially impacting on student ratings. Thus, a total of 25 studies were included in the analysis.

### Description of included studies

Details on study year, location, design, methods, results and study quality are summarised in Additional file 2: Table S1 in the online supplement of this article. Sixteen studies, including all nine research projects completed up until the year 2000, were conducted in the US. More recent studies were conducted in European countries (n = 6), including Germany, the Netherlands, the United Kingdom, and Sweden. Two studies were done in Canadian medical schools, and one paper reported the results of a research collaboration involving Canada and the Netherlands. Despite the small number of included trials, there was a trend for increasing research output in recent decades: While the 1980s only saw two publications on the use of student ratings for course evaluation in undergraduate medical education, this number slightly increased to three in the 1990s and jumped to nine in the 2000s. In the short period from 2010 to 2013 alone, eight more reports have been published. We found two qualitative studies, and eight of the 23 quantitative trials used a randomised study design.

### Results of qualitative studies

Two qualitative studies from the US [5] and Germany [12] including small samples (n = 24 and n = 17, respectively) addressed medical students’ attitudes towards course evaluations and approaches to completing evaluation forms. Think-aloud interviews revealed that items on evaluation forms were ambiguous for some students, leading to student ratings being based on unique or unexpected definitions of the terms used. For example, one student felt a course provided a ‘solid foundation for future learning’ if the course material matched the content of Board review books [5]. With regard to overall course ratings, students tended to rely on their ‘gut feelings’ rather than using objective benchmarks of course quality [12]. As a result, overall course ratings appeared to be mainly influenced by student satisfaction with teaching and exam difficulty [12]. It is unclear to what extent satisfaction reflects factors underpinning high quality teaching.

### Results of quantitative studies

The MERSQI score for quantitative studies ranged from 7.0 to 11.5 (mean 8.7 ± 1.1 out of a maximum of 18). Studies were grouped into four broad categories (see Table 1) with some studies relating to more than one category: The first set of studies (n = 6) assessed the extent to which student characteristics including gender, initial interest and final exam performance affect course ratings provided by students. The second set of studies (n = 6) focussed on the association between teaching structure/process/content and course ratings. Surprisingly few studies (n = 3) investigated the predictive value of student satisfaction with end-of-course examinations for overall course ratings while the impact of the process of evaluation (including timing and design of the evaluation tool) on student ratings was assessed in 13 studies. In the following sections, results related to these four categories will be presented in turn.

**Table 1:** **Main findings of quantitative research (see text for details)**

Student characteristics	Structure, process and content of teaching	Examinations	Evaluation process
• Gender: Female students tend to provide more positive ratings (2 studies).	• Procedural aspects of teaching: Course organisation, effective communication of learning objectives and high staff responsiveness are associated with higher overall ratings (1 study).	• Satisfaction with examinations: Students who are more satisfied with end-of-course examinations tend to provide more favourable course ratings (2 studies).	• Timing of data collection: Course ratings provided retrospectively (i.e., up to one year after a course) can be less favourable (2 studies) or slightly more favourable (1 study) than ratings provided at the end of a course.
• Initial interest: Students who are more interested in course content tend to provide more positive ratings (2 studies).	• Didactic methods: Provision of high quality feedback predicts overall ratings (1 study).	• Blueprint availability: Availability of an examination blueprint improves overall course ratings (1 study).	• Data collection tool: As opposed to paper/pencil evaluations, online evaluations yield lower response rates (1 study) but slightly more favourable ratings (1 study).
• Performance level: High-performing students tend to provide more positive ratings (2 studies).	• Presentation format: Live lectures receive more favourable ratings than identical, videotaped lectures (1 study).		• Response rate / selection bias: Data obtained in a mandatory evaluation procedure are no different from data obtained in a voluntary setting (1 study). High-achievers might (1 study) or might not (1 study) be over-represented in student samples self-selecting to participate.
	• Attendance: Mandatory seminars receive more positive overall ratings than lectures with voluntary attendance (1 study). Students voluntarily attending lectures tend to provide more positive ratings than non-attendees (1 study).		• Point of reference: Asking students to predict how their peers would rate a course produces the same results as obtaining individual ratings but requires fewer participants to get stable results (2 studies).
	• Teacher attitudes: Negative teacher attitudes towards a course negatively influence student ratings (1 study).		• Design of rating scales: Positively phrased items and scales with the positive anchor on the left and no labels on intermediate scale options yield the most favourable ratings (3 studies).

#### Student characteristics

Two studies assessed the impact of gender on student ratings. In a cohort of 308 third-year students from Manchester, female gender was predictive of more positive course ratings [13]. A more recent study from the Netherlands and Canada reported a similar finding in first- and third-year students [14]. Two studies from Germany covering all years of undergraduate medical education obtained student ratings of their initial interest in course content. In one study, these were significantly and strongly correlated to post-course ratings of didactic quality [15], and the other study found a strong correlation with overall ratings obtained after course attendance [16].

Two similar studies conducted before 1980 at the Medical University of South Carolina reported positive correlations (r ≈ 0.4) between student performance in a final exam and overall ratings of an anatomy course [17,18]. One of these studies also investigated whether the timing of data collection (i.e. before or after the exam) influenced student ratings and did not find an effect [17]. However, generalisability of this result is limited by the fact that the exam was perceived as only moderately difficult by students.

In summary, female gender, greater initial interest and higher final exam scores are associated with more positive overall course ratings.

#### Structure, process and content of teaching

In one study including 84 first- and 64 third-year students from Texas A&M University, factor analyses of five different questionnaires (15–24 items) revealed that positive overall ratings were associated with positive assessments of course organisation, effective communication of learning objectives and good staff responsiveness [19]. Another study identified receiving high quality feedback as an independent predictor of overall student ratings of a third-year clerkship [20]. In a group of 40 second-year students at the University of California Medical Centre, those randomised to attend a live lecture provided more favourable ratings than those watching a video recording of the same lecture [21]. One German study found that mandatory seminars received more positive overall ratings than lectures with voluntary attendance [15]. At the same time, students voluntarily attending lectures tended to provide more positive ratings than non-attendees in this [15] and another [17] study. Finally, one Swedish study suggested that teacher attitudes towards a course might frame student appraisal of the course [22].

In summary, teaching format and exposure to teaching impact on student ratings with students voluntarily attending lectures providing more positive ratings. Unfortunately, interest in the subject matter or initial student motivation was not assessed in these studies.

#### Satisfaction with exams

One recent study including a total of 750 first- and third-year medical students from Canada and the Netherlands concluded that student satisfaction with end-of-course exams predicted overall course ratings [14]. Similar results were reported in another Canadian study involving 800 first- and second-year students [7]. The same research team also investigated the effect of making an exam blueprint (outlining exam content) available to students on evaluation results and found that blueprint availability increased overall course ratings [23]. In summary, student satisfaction with exams appears to be a consistent predictor of course ratings.

#### Process of evaluation

Studies investigating the association between the timing of data collection and student ratings have yielded conflicting results: One early study from Washington University (involving 104 second-year students) found that ratings provided at the end of a course were generally lower than ratings provided during the course [24]. A similar trend was observed over longer periods (i.e., one year) in another trial conducted at the same medical school [3]. Contrary to this, a more recent study carried out at the University of Pennsylvania Medical School (involving 304 first- and second-year students) reported that student ratings increased as a function of elapsed time (in weeks) after a teaching event. However, the average size of this effect was negligible (Cohen’s d = 0.06) [25].

Despite most medical schools now using online platforms to collect evaluation data, we identified no more than two studies assessing the way in which data collection format (online versus paper and pencil) impacts on overall course ratings. One older study [26] found lower response rates in the online approach (19% vs. 41%) but no significant differences in ratings. However, the study might have been underpowered to detect such differences. Another more recent study [27] analysed approximately 5,000 evaluation forms and found that overall ratings were significantly more positive in the online condition; however, the effect size of this difference was small (Cohen’s d = 0.18).

As participation in evaluation activities is not mandatory at all universities, self-selection of students providing course ratings might produce biased samples. As a consequence, the reliability and validity of course ratings would depend on response rates. While no study has directly assessed this potential association, three reports provide indirect evidence that selection bias might not pose a major threat to the validity of student ratings. Purkiss suggested that low-achievers might be over-represented in the subgroup of students who voluntarily completed evaluation forms. However, data obtained from just under 700 students in three consecutive academic years at the University of Michigan Medical School did not support this hypothesis. Instead, in three out of 22 courses, students completing evaluation forms had achieved moderately higher exam scores than their peers (effect size calculated as Cohen’s d: 0.37-0.58) [28]. In an earlier study from Washington University, about 150 first- and second-year students were randomised to a mandatory or a voluntary evaluation group. Data obtained from both groups were largely similar [29]. A third study from the same decade found no difference in performance levels between students who responded to a posted evaluation survey and those who did not [17]. Although these results suggest that self-selection of students does not produce severely biased samples, performance level is but one student characteristic, and other characteristics potentially impacting on the willingness to participate (thus generating selection bias) have not been studied.

More recently, new ways of dealing with low response rates in medical education course evaluations have been identified. Two papers from the Netherlands and Canada using the same methodology showed that asking students to predict how their peers would have rated a course resulted in similar results as asking them to provide their own ratings [30]. The prediction-based method required fewer respondents to produce stable results; in addition, it was more robust against bias than individual ratings [14].

Finally, three studies investigated how the design of rating scales impacts on student ratings. Two of these [31,32] were conducted by the same group at Wisconsin Medical School, and none used data collected after the year 2000. The principal findings of these studies were:

- Rating scales with the most positive option on the left produced more favourable mean ratings with smaller variance than scales with positive anchors on the right [31].
- Only labelling the extreme right and left poles of a scale yielded more favourable ratings than labelling all scale options [32].
- Negatively phrased items were associated with lower scale reliability and were less sensitive to change over time [33].

In summary, the effects of timing and response rates of evaluation activities are ambiguous, and few studies from the US provide evidence of a significant impact of rating scale design on evaluation results.

## Discussion

To our knowledge, this is the first systematic review of factors influencing student course ratings with a specific focus on undergraduate medical education. Earlier reviews considered original research in higher education settings other than medical education or focussed on other dependent variables such as teaching effectiveness [2]. Given the distinctive features of medical education within higher education and the wide-spread use of overall course ratings in evaluations, the present review aimed to provide an up-to-date overview of factors impacting on student ratings. Medical educators should be aware of these factors when designing data collection tools and interpreting overall course ratings.

The main finding of this review is that high-quality research on such factors influencing overall ratings is scarce and to some extent equivocal. The mean MERSQI score of 8.7 for quantitative studies is evidence of considerable room for improvement in this area. Although various research questions have been raised in the past 40 years, we found a maximum of four studies – and usually no more than one or two – addressing the same question. There is no uniform standard in reporting data collection tools and results, and rarely has the same questionnaire or rating scale been used in two different studies. Generalisability of quantitative results is further limited by the fact that most studies only involved students in one particular year at one particular medical school with sample sizes ranging from 40 [21] to 1100 [33] students and response rates ranging from 36.7% [16] to 94.4% [32]. The majority of quantitative studies were purely observational, and additional influencing factors were not reported and/or controlled for in most studies. For example, studies assessing the impact of student satisfaction with exams on overall ratings should also report the item characteristics (i.e., item difficulty and discriminatory power) of the exams used. Likewise, studies assessing the association between lecture attendance and overall ratings should also report performance levels and motivation of attendees and non-attendees. The very same factors causing students to attend or not to attend a lecture could well have a bearing on course ratings provided by these students [34].

Given these limitations, the results of this systematic review must be interpreted with caution. Its most consistent findings relate to student characteristics and student satisfaction with exams in that female and more motivated students, high-achievers and those who are more satisfied with exams tend to provide more positive course ratings. Any selection procedure favouring these groups might entail inflated ratings.

### Suggestions for future research and evaluation practices

The paucity of high-quality research into factors threatening the validity of student ratings may be one reason why some programme directors pay more attention to occasional qualitative feedback (provided by a highly selected student sample) than to aggregated quantitative data [35]. While free-text comments and discussions with students should always be an integral part of evaluation activities, overall course ratings might be more suitable for comparative evaluations and performance-guided resource allocation within medical schools. However, in order to be used for this purpose, data collection and interpretation must be highly standardised – both within and across different medical schools. While increasing response rates would seem the most effective way to avoid selection bias, it is also difficult to achieve. In this regard, recent reports of ‘prediction-based’ data collection tools producing reliable results in small student samples seem promising [14,30].

A sophisticated approach to interpreting evaluation data requires an in-depth understanding of the psychological mechanisms underlying the effects described in this review. While a comprehensive overview of all mechanisms potentially affecting student ratings is beyond the scope of this review, some of the most salient effects (e.g., Reward-retaliation effect [36], Recency effect [37], Primacy effect [38] and Generosity error [39]) are being discussed the original articles included in this review [7,17,25,32]. Psychological factors impacting on student ratings need to be considered when designing data collection instruments, underscoring the need to consult experts in psychometrics in the process [2].

The considerable uncertainties regarding the reliability and validity of student ratings compiled in this review once again highlight the importance of using multiple sources of evidence when evaluating undergraduate medical curricula [2].

### Strengths and limitations

Overall course ratings reflect student satisfaction with various facets of teaching (structural and procedural aspects, learning outcome, and teacher performance [1]). We cannot comment on the contribution of each of these to overall student ratings. Consistent with the aim of this review, we did not limit our analysis to studies focussing on only one specific dimension of teaching quality. Instruments that were designed to address specific areas (e.g. the ‘Undergraduate Clinical Education Environment Measure’ [40] for procedural aspects, the ‘CSA gain tool’ [41] for learning outcome and the ‘Student Evaluation of Teaching in Outpatient Clinics’ questionnaire [42] for individual teacher performance) were beyond the scope of this review. These tools do not produce overall ratings but distinct results for various aspects of teaching quality. Similarly, studies on student self-ratings rather than overall course ratings were excluded. While this might have decreased the variety of studies included in our review, it allowed us to focus on one specific research question which is a particular strength of this review.

Our interpretation of the data was hampered by the fact that we did not have access to the original questionnaires used in most studies. Seeing the exact wording of evaluation questions might have enabled us to judge which (if any) questionnaires provided benchmarks of high quality education that would have helped students to arrive at a more objective rating. In this case, high student satisfaction with teaching might in fact reflect aspects of high quality teaching. However, this assertion is speculative and should be addressed in future studies.

Finally, despite our efforts to identify all relevant studies by searching three different databases and using a fairly large number of search terms, we might have missed relevant studies, particularly if factors influencing student ratings were only addressed in secondary or subgroup analyses not mentioned in the abstract. We did not consult a reference librarian to support our literature search and did not search all available databases. However, we are confident that we were able to identify the vast majority of relevant studies as all journals publishing medical education research are indexed in at least one of the databases searched. For example, scanning Web of Science after completing searches in Pubmed and PsycInfo produced 61 additional unique citations only one of which met our inclusion criteria.

## Conclusion

Student ratings of courses in undergraduate medical education reflect student satisfaction with various facets of teaching. This systematic review identified a number of factors impacting on overall course ratings. Depending on the underlying construct of high-quality teaching, these factors might act as confounders, thus threatening the validity of evaluation results. Influencing factors were related to student characteristics, exposure to teaching, satisfaction with examinations and the evaluation procedure itself. Due to the heterogeneity and methodological limitations of included studies, no firm conclusions can be drawn from this review. Medical educators need to be aware of various factors potentially impacting on student ratings when developing data collection instruments and interpreting evaluation results. More research into the control of potential confounders and the development of robust evaluation instruments is warranted.

## Additional files

Additional file 1: **PRISMA Checklist for this systematic review.** This checklist indicates where relevant information on the preparation of this systematic reviews can be found in the text. Page numbers refer to the final Word version of the manuscript submitted on February 16, 2015. Additional file 2: Table S1. Characteristics and main results of included studies (sorted by year of publication in ascending order). This table gives a summary of all included studies. It contains information on the year(s) the research was done, the country/countries where the study was conducted, the study design, the study sample and the evaluation tool used, as well as the main results and the MERSQI Score.

## References

[1] Gibson KA, Boyle P, Black DA, Cunningham M, Grimm MC, McNeil HP (2008). Enhancing Evaluation in an Undergraduate Medical Education Program. Acad Med.

[2] Berk RA (2013). Top five flashpoints in the assessment of teaching effectiveness. Med Teach.

[3] Scott CS, Hunt DD, Greig LM (1986). Changes in course ratings following clinical experiences in the clerkship years. J Med Educ.

[4] Kogan JR, Shea JA (2007). Course evaluation in medical education. Teach Teach Educ.

[5] Billings-Gagliardi S, Barrett SV, Mazor KM (2004). Interpreting course evaluation results: insights from thinkaloud interviews with medical students. Med Educ.

[6] Haidet P, Stein HF. The role of the student-teacher relationship in the formation of physicians. The hidden curriculum as process. J Gen Intern Med. 2006; 21 Suppl 1:S16–20. 10.1111/j.1525-1497.2006.00304.x PMC1484835 16405704

[7] Woloschuk W, Coderre S, Wright B, McLaughlin K (2011). What factors affect students’ overall ratings of a course?. Acad Med.

[8] Soemantri D, Herrera C, Riquelme A (2010). Measuring the educational environment in health professions studies: a systematic review. Med Teach.

[9] Raupach T, Brown J, Herbec A, Brose L, West R (2014). A systematic review of studies assessing the association between adherence to smoking cessation medication and treatment success. Addiction.

[10] Moher D, Liberati A, Tetzlaff J, Altman DG (2009). Preferred reporting items for systematic reviews and meta-analyses: the PRISMA statement. BMJ.

[11] Reed DA, Cook DA, Beckman TJ, Levine RB, Kern DE, Wright SM (2007). Association between funding and quality of published medical education research. JAMA.

[12] Schiekirka S, Reinhardt D, Heim S, Fabry G, Pukrop T, Anders S (2012). Student perceptions of evaluation in undergraduate medical education: A qualitative study from one medical school. BMC Med Educ.

[13] Dornan T, Arno M, Hadfield J, Scherpbier A, Boshuizen H (2006). Student evaluation of the clinical ‘curriculum in action’. Med Educ.

[14] Schonrock-Adema J, Lubarsky S, Chalk C, Steinert Y, Cohen-Schotanus J (2013). What would my classmates say? An international study of the prediction-based method of course evaluation. Med Educ.

[15] Berger U, Schleussner C (2003). Strauss B: [Comprehensive evaluation of medical teaching – a task for the psychosocial disciplines?]. Psychother Psychosom Med Psychol.

[16] Raupach T, Schiekirka S, Munscher C, Beissbarth T, Himmel W, Burckhardt G, et al. Piloting an outcome-based programme evaluation tool in undergraduate medical education. GMS Z Med Ausbild 2012, 29:Doc44. 10.3205/zma000814 PMC3374140 22737199

[17] Canaday SD, Mendelson MA, Hardin JH (1978). The effect of timing on the validity of student ratings. J Med Educ.

[18] Mendelson MA, Canaday SD, Hardin JH (1978). The relationship between student ratings of course effectiveness and student achievement. Med Educ.

[19] Sadoski M, Sanders CW (2007). Student Course Evaluations: Common Themes across Courses and Years. Med Educ Online.

[20] Torre DM, Simposon D, Bower D, Redlich R, Plma-Sisto P, Lund MR (2006). Learning Activities and Third-Year Medical Student Ratings of High Quality Teaching Across Different Clerkships. Med Educ Online.

[21] Leamon MH, Servis ME, Canning RD, Searles RC (1999). A comparison of student evaluations and faculty peer evaluations of faculty lectures. Acad Med.

[22] Lynoe N, Juth N, Helgesson G (2012). Case study of a framing effect in course evaluations. Med Teach.

[23] McLaughlin K, Coderre S, Woloschuk W, Mandin H (2005). Does blueprint publication affect students’ perception of validity of the evaluation process?. Adv Health Sci Educ Theory Pract.

[24] Irby DM, Shannon NF, Scher M, Peckham P, Ko G, Davis E (1977). The use of student ratings in multiinstructor courses. J Med Educ.

[25] McOwen KS, Kogan JR, Shea JA (2008). Elapsed time between teaching and evaluation: does it matter?. Acad Med.

[26] Paolo AM, Bonaminio GA, Gibson C, Partridge T, Kallail K (2000). Response rate comparisons of e-mail- and mail-distributed student evaluations. Teach Learn Med.

[27] Burton WB, Civitano A, Steiner-Grossman P (2012). Online versus paper evaluations: differences in both quantitative and qualitative data. J Comput High Educ.

[28] Purkiss J (2012). Course evaluation respondents: are ‘low-performing retaliators’ really over-represented?. Med Educ.

[29] Carline JD, Scher M (1981). Comparison of course evaluations by random and volunteer student samples. J Med Educ.

[30] Cohen-Schotanus J, Schonrock-Adema J, Schmidt HG (2010). Quality of courses evaluated by ‘predictions’ rather than opinions: Fewer respondents needed for similar results. Med Teach.

[31] Albanese M, Prucha C, Barnet JH, Gjerde CL (1997). The effect of right or left placement of the positive response on Likert-type scales used by medical students for rating instruction. Acad Med.

[32] Albanese M, Prucha C, Barnet JH (1997). Labeling each response option and the direction of the positive options impacts student course ratings. Acad Med.

[33] Stewart TJ, Frye AW (2004). Investigating the use of negatively phrased survey items in medical education settings: common wisdom or common mistake?. Acad Med.

[34] Pabst R, Nave H, Rothkotter HJ, Tschernig T (2001). Evaluation of the medical curriculum: why, when, by whom and for whom should questionnaires be used. Eur J Morphol.

[35] Hendry GD, Cumming RG, Lyon PM, Gordon J (2001). Student-centred course evaluation in a four-year, problem based medical programme: Issues in collection and management of feedback. Assess Eval Higher Educ.

[36] Marsh HW, Fleiner H, Thomas CS (1975). Validity and Usefulness of student evaluations of instructional quality. J Educ Psychol.

[37] Constable KA, Klein SB (2005). Finishing strong: Recency effects in juror judgments. Basic Appl Soc Psych.

[38] Leventhal L, Turcotte SJC, Abrami PC, Perry RP (1983). Primacy/recency effects in student ratings of instruction: A reinterpretation of gain–loss effects. J Educ Psychol.

[39] Mehrens W, Lehmann I (1984). Measurement and Evaluation in Education and Psychology.

[40] Strand P, Sjoborg K, Stalmeijer R, Wichmann-Hansen G, Jakobsson U, Edgren G (2013). Development and psychometric evaluation of the Undergraduate Clinical Education Environment Measure (UCEEM). Med Teach.

[41] Schiekirka S, Reinhardt D, Beissbarth T, Anders S, Pukrop T, Raupach T (2013). Estimating learning outcomes from pre- and posttest student self-assessments: a longitudinal study. Acad Med.

[42] Zuberi RW, Bordage G, Norman GR (2007). Validation of the SETOC instrument – Student evaluation of teaching in outpatient clinics. Adv Health Sci Educ Theory Pract.

