# Supplementary material for: A systematic review of factors influencing student ratings in undergraduate medical education course evaluations
Source: BMC Med Educ. 2015 Mar 5;15:30. doi: 10.1186/s12909-015-0311-8 (PMC4391198; doi:10.1186/s12909-015-0311-8)
Supplement: Additional file 2: Table S1. — Characteristics and main results of included studies (sorted by year of publication in ascending order). This table gives a summary of all included studies. It contains information on the year(s) the research was done, the country/countries where the study was conducted, the study design, the study sample and the evaluation tool used, as well as the main results and the MERSQI Score. [file 12909_2015_311_MOESM2_ESM.pdf]

Table S1: Characteristics and main results of included studies (sorted by year of publication in ascending order).

Study	Year*	Country	Randomised	Quantitative	Qualitative	Study sample	Evaluation tool	Main results	MERSQI Score**
[1]	1975	USA	X	X		104 second-year students enrolled in a 30-hour psychopathology course at Washington University (13 different lecturers). On each occasion, only ¼ of all students were asked to complete evaluation forms. Overall response rate ~80%	13 items with 6-point scales, mainly directed at teaching skills	Post-course ratings were significantly lower than in-course ratings.<br>Daily ratings for the course stabilised over time.	10
[2]	before 1978	USA	X	X		Of 158 students enrolled in an anatomy course at the Medical University of South Carolina, 113 provided data (45 before an exam, 42 after an exam, and 26 out of 71 who were sent the form 3 weeks later). Twenty students were excluded from the analysis; thus, total response rate was 58.9% (93/158).	31 items with 5-point Likert scales; the first 20 items assessed attitudes (satisfaction) and produced a sum score (max. 100 for the most positive rating)	sum score = 81.2 ± 7.8<br>High-achievers provided more positive ratings than low-achievers (r between performance and evaluation ratings: 0.42), but time of rating did not affect results; no interaction between achievement and timing.<br>Lecture attendees had higher exam scores and provided better evaluation ratings (potential confounding by recency effects).<br>No sign. difference in performance levels between respondents and non-respondents (post-exam group only).	9.5
[3]	before 1978	USA		X		124-140 students enrolled in an anatomy course at the Medical University of South Carolina<br>Response rate 79-90%	67 items with 5-point Likert scales; the first 20 items assessed attitudes (satisfaction) and produced a sum score (max. 100 for the most positive rating)	sum score = 77.9 ± 9.6<br>Correlation between course exam performance and evaluation ratings: 0.33<br>Correlation between grade point average and evaluation ratings: 0.13 (n.s.)	9
[4]	1979	USA	X	X		71 out of 101 (70.3%) and 86 out of 148 (58.1%) first-year students in the (1) Molecular and Cellular Biology or (2) Psychopathology course at Washington University. In both courses, 25% of students were randomly appointed to complete a mandatory evaluation. All other students were invited to complete a voluntary evaluation.	20 (course 1) or 13 (course 2) items on 6-point scales	Data obtained from the ‘voluntary’ and the ‘mandatory’ samples were largely similar.<br>No clear pattern on differences in variances (could be expected to be higher in voluntary samples as more extreme ratings might be given).	9.5
[5]	1981-1985	USA		X		Participants were selected from 175 students enrolled in 28 different 1st- or 2nd-year courses at the University of Washington School of Medicine.<br>Response rates for end-course ratings: 90% for first-year courses and 83% for second-year courses<br>Response rates for retrospective ratings: 84% for first-year courses and 67% for second-year courses	2 items (amount learned and overall rating) on 6-point scales	Retrospective ratings provided 1 year after the course were less favourable then direct ratings.<br>Retrospective ratings provided 2 years after the course were stable compared to ratings provided 1 year after the course.	9

[6]	1995-1996	USA	X	X		132 (94% response rate) / 119 (85% response rate) out of 140 second-year students enrolled in two different courses at the University of Wisconsin Medical School	13 items on 5-, 6- or 7-point scales. The positive option was either on the left- or on the right-hand side.	Scales with positive anchors on the left produced significantly more favourable ratings with less variance than scales with positive anchors on the right.<br>In a course with more positive overall ratings, the primacy effect was stronger for SDs than for means and also stronger for scales with fewer options.<br>In a course with less positive overall ratings, the primacy effect was stronger for mean than for SDs and also stronger for scales with more options.	10
[7]	1995-2000	USA		X		Approximately 1100 first-year students enrolled at the University of Texas<br>Response rate not reported	Medical School Learning Environment Survey (MSLES) with seven scales: 55 items on 5-point scales<br>Students completed the MSLES twice (at the beginning and the end of the first year).	When all positive-items within a scale were collapsed into one new scale, this yielded higher mean scores than a sub-scale only containing negative-item scales.<br>Negatively phrased items were associated with lower scale reliability and were less sensitive to change over time.	7.5
[8]	before 1997	USA	X	X		135 out of 143 (94.4%) first-year students at Wisconsin Medical School	3 items on 6-point scales; four different versions of the mailed questionnaire were used:<br>a) Positive option on the left, labels only on the extreme poles<br>b) Positive option on the left, all options labelled (outstanding, excellent, very good, good, fair, poor)<br>c) Positive option on the right, labels only on the extreme poles<br>d) Positive option on the right, all options labelled	All but one scales yielded similar results; however, scale d) produced the least favourable ratings as students were driven towards more negative options by the predominantly positive labels.<br>In general, placing positive labels on the left and only labelling the extreme poles (instead of all options) yielded more favourable ratings.	10
[9]	1998	USA	X	X		40 second-year students (20 for live and 20 for videotaped lectures) and 31 faculty at the University of California Medical Centre	11 items on 5-point scales addressing teacher characteristics, learning material and effectiveness of the lecture	Overall, student ratings were slightly (non-significantly) less favourable then peer faculty ratings.<br>Students rated live lectures more favourably than identical videotaped lectures.	9
[10]	1998-2002	Canada		X		123 (before blueprint publication; response rate 53.7%) and 114 (after publication; response rate 55.1%) students enrolled in a renal course at the University of Calgary	a) Exam performance<br>b) Satisfaction with the exam (4 items on 5-point scales)<br>c) Satisfaction with the course (1 item on a 5-point scale)	Following publication of the exam blueprint, students were slightly more satisfied with both the exam and (non-significantly) the course itself.	8.5
[11]	1999-2002	Germany		X		No information on sample size (169 lectures, 288 seminars)	13 to 15 items on 6-point scales	Factor analysis produced two factors in the 13- to 15-item tool; the first factor (‘didactics’) was correlated to initial interest (r = 0.59).<br>Higher lecture attendance (>80%) was associated with better ratings than less frequent attendance (<80%; effect size of the difference ε = 0.44)<br>Mandatory seminars received better ratings than lectures with voluntary attendance.	8
[12]	before 2000	USA		X		34 out of 83 (41%) and 15 out of 81 (19%) fourth-year students completing paper and online forms, respectively	62 items on 5-point scales addressing different clearkships	1) Response rate: online 19%; paper 41%; more omitted items in online forms<br>2) Online (e-mailed) forms were returned more quickly than mailed paper forms.<br>3) no significant differences in ratings between online and paper forms.	8
[13]	2001	USA		X		110 third-year students enrolled in 4 different clerkships (pediatrics, surgery, obstetrics/gynecology and family medicine) at the Medical College of Wisconsin.<br>Each student recorded approximately 100 encounters.	‘Patient encounter questionnaire’: 6 items to be completed on a personal digital assistant (most of them dichotomous).<br>The dependent variable was ‘overall teaching quality’ (rated as outstanding / very good / good / marginal / unsatisfactory).	Exposure to most learning activities was associated with better overall ratings; the strongest association was observed with receiving high quality feedback. This was the only independent predictor for all clerkships.	7

[14]	2001-2004	UK		X		308 third-year students enrolled at Manchester University (PBL curriculum); response rates for individual dimensions of teaching ranged between 79 and 91%.	Web-based form, 13 items on 7-point Likert scales; collapsed into 4 dimensions: conditions for learning, quality of instruction, real patient learning, curriculum coverage	Exam results were correlated with gender and real patient learning.<br>In a multivariate analysis with real patient learning as the dependent variable, the other 3 dimensions of teaching as well as gender and exam results were significant predictors.<br>In another model with end-of-year exam results as the dependent variable, only mid-year exam results and (to a lesser extent) real patient learning was predictive.<br>Associations were stronger for women than men.	8
[15]	before 2004	USA			X	24 self-selected second-year students enrolled in the Mind, Brain and Behaviour course at the University of Massachusetts Medical School	13 items on 4- to 5-point scales (plus a few teacher ratings).<br>Think-aloud interviews were done while students completed these forms.	Evaluation items were ambiguous for some students.<br>Student ratings were based on unique or unexpected criteria.<br>The lower end of the rating scale tended to be avoided.<br>Exams were not mentioned by students as potential confounders of overall ratings.	—
[16]	2004-2005	USA		X		84 first-year and 64 third-year students enrolled in 5 specialty courses at Texas A&M University<br>Response rate 100% (mandatory)	Course-specific forms with 15-24 items on 5-point scales and 1 overall rating on a 4-point scale.<br>Evaluation forms were completed either at the end of an entire course of at the end of a rotation within a course.	The following items were associated with better overall ratings:<br>a) Administrative aspects including course organization<br>b) Clearly communicated goals<br>c) Instructional staff responsiveness<br>Similar loadings were observed in different courses.	8
[17]	2002-2009	USA		X		Third-year students at Albert Einstein College of Medicine, New York. No information on the number of students involved; 2141 paper and 2732 online evaluation forms were analysed.	23 items on 5-point scales<br>Paper forms were used in 2002-2005; online forms were used in 2005-2009	Response rate: paper 95%, online 60-85%<br>Factor analysis for both versions yielded similar results.<br>Aggregate scores were higher (i.e. more positive) after switching from paper to online (effect size d = 0.18).	8
[18]	2006	USA		X		304 students attending a total of 531 events at the University of Pennsylvania Medical School; response rate ~90%	Session satisfaction rating on a 5-point scale via a web tool	With more elapsed weeks, quality mean ratings increased and variability decreased; effect sizes were small (around 0.06).	8
[19]	2006-2007	The Nether-lands	X	X		Study 1: 380 first-year students; response rates: opinion condition 79%; prediction condition 60%<br>Study 2: 450 first-year students; response rates: opinion condition 88%; prediction condition a 76%; prediction condition b 70%<br>All students were enrolled in the 10-week ‘Bodily functions and homeostasis’ course at the University Medical Centre Groningen	Paper evaluation forms (9 items on 4-point scales) to be completed after the final course exam	Both prediction-based methods required fewer respondents than the opinion-based method. Informed prediction required the smallest sample size.<br>Outcomes produced by all methods were fairly similar, but prediction-based methods produced less extreme results. This central tendency was more pronounced for items with more extreme ratings in the opinion condition.	9.5 / 10
[20]	2007-2008	Canada		X		391 out of 606 (64.5%) first- and 234 out of 416 (56.3%) second-year students enrolled in seven courses at the University of Calgary	20 items on teaching and 5 items on exams, 1 overall rating (all on 5-point scales)<br><br>Online evaluations were closed before students were informed about exam results.	Four factors were identified (loaded on by 11 out of 25 items):<br>a) Exams (fairness and alignment with course objectives)<br>b) Small-group learning<br>c) Basic science teaching<br>d) Teaching diagnostic approaches<br>Together, these explained 50% of the variance.<br>Overall ratings were most strongly associated with ratings related to the exam. In the second year, exams were the only predictors of overall ratings.	7.5

[21]	2008-2009	Sweden		X		Students enrolled in a course of philosophy in medicine at Karolinska Institutet before (n = 96) and after (n = 79) a curricular change reducing the course from 4 to 2 days<br>Response rate not reported	2 items on 5-point scales (assessing effectiveness of and satisfaction with the course) plus free text comments	Student ratings on both dimensions were more positive after the course had been shortened. The authors interpret their findings as evidence of a framing effect: Teachers’ frustration with curricular change might have influenced student ratings.	8
[22]	2008-2010	USA		X		684 students enrolled in 22 pre-clinical courses at Michigan Medical School<br>Response rate not reported	Course and teacher evaluations (no specific information on data collection tools)	a) Teacher evaluation: In 6 courses, participants had higher exam scores than non-participants (effect size d 0.35-0.55).<br>b) Course evaluation: In 3 courses, participants had higher exam scores than non-participants (effect size d 0.37-0.58).	7
[23]	2010-2011	Canada & The Nether-lands	X	X		198 out of 210 (94.3%) first-year students at McGill University (Montreal) as well as 270 out of 371 (72.8%) first-year and 270 out of 385 (70.1%) third-year students at the University Medical Centre Groningen	Paper questionnaire containing 10 items on 4-point Likert scales, to be completed directly after the end-of-course examination<br>Half of all students in each cohort were asked to provide their own ratings; the other half were asked to guess what their fellow students would say (percentages for each of the 4 scale options).<br>Additional items on gender, perceived performance level, expected exam results, mood after exam completion	The prediction-based method required fewer respondents than the opinion-based method.<br>Outcomes produced by the two methods were fairly similar, but overall, the prediction-based method produced less extreme results.<br>Prediction-based outcome data were more robust against bias; individual ratings were more positive in students who were female and more satisfied with the exam.	11.5
[24]	2011	Germany		X		573 out of 977 students in years 3-5 at Göttingen Medical School; response rates for individual teaching modules: 36.7-75.4%	a) Motivation survey (3 items on 6-point scales) at the start of each module<br>b) Traditional evaluation form with 6 items on 6-point scales (after each module)<br>c) Performance gain calculated from repetitive self-assessments (before and after each module). Average values for 15 learning objectives per teaching module	The traditional tool and the performance gain tool produced different module rankings.<br>Motivation ratings obtained before module attendance were positively correlated with evaluation ratings obtained after the modules.<br>All items on the traditional tool were highly correlated with each other; there was hardly any correlation with performance gain results.	8.5
[25]	2011	Germany			X	17 self-selected students in years 3-4 at Göttingen Medical School	Does not apply	Student remarks were related to 4 distinct themes (teaching quality, perceptions of evaluation, data collection tools, evaluation consequences).<br>Student ratings are mainly based on ‘gut feelings’ rather than objective benchmarks. Overall ratings are mainly influenced by student satisfaction with teaching and exam difficulty. Students are more satisfied with teaching if they got the feeling to have learned something.<br>Low response rates may be due to evaluation overload or a lack of feedback following evaluation.<br>Students preferred evaluations to occur after end-of-course exams. They also preferred online over paper evaluations and open questions / discussions over scaled questions.	—

*Year refers to the time when the study was conducted, not year of publication. Please see the reference list for year of publication.

**MERSQI Score was derived from two independent ratings for each study. Differences between the two raters were resolved by discussion.

Qualitative studies did not receive a MERSQI rating. One paper ([20]) reported findings of two different studies. MERSQI scores for these two studies are displayed separately.

References

1. Irby DM, Shannon NF, Scher M, Peckham P, Ko G, Davis E: **The use of student ratings in multiinstructor courses.** *J Med Educ* 1977, **52:**668-673.
2. Canaday SD, Mendelson MA, Hardin JH: **The effect of timing on the validity of student ratings.** *J Med Educ* 1978, **53:**958-964.
3. Mendelson MA, Canaday SD, Hardin JH: **The relationship between student ratings of course effectiveness and student achievement.** *Med Educ* 1978, **12:**199-204.
4. Carline JD, Scher M: **Comparison of course evaluations by random and volunteer student samples.** *J Med Educ* 1981, **56:**122-127.
5. Scott CS, Hunt DD, Greig LM: **Changes in course ratings following clinical experiences in the clerkship years.** *J Med Educ* 1986, **61:**764-766.
6. Albanese M, Prucha C, Barnet JH, Gjerde CL: **The effect of right or left placement of the positive response on Likert-type scales used by medical students for rating instruction.** *Acad Med* 1997, **72:**627-630.
7. Stewart TJ, Frye AW: **Investigating the use of negatively phrased survey items in medical education settings: common wisdom or common mistake?** *Acad Med* 2004, **79:**S18-20.
8. Albanese M, Prucha C, Barnet JH: **Labeling each response option and the direction of the positive options impacts student course ratings.** *Acad Med* 1997, **72:**S4-6.
9. Leamon MH, Servis ME, Canning RD, Searles RC: **A comparison of student evaluations and faculty peer evaluations of faculty lectures.** *Acad Med* 1999, **74:**S22-24.
10. McLaughlin K, Coderre S, Woloschuk W, Mandin H: **Does blueprint publication affect students' perception of validity of the evaluation process?** *Adv Health Sci Educ Theory Pract* 2005, **10:**15-22.
11. Berger U, Schleussner C, Strauss B: **[Comprehensive evaluation of medical teaching -- a task for the psychosocial disciplines?].** *Psychother Psychosom Med Psychol* 2003, **53:**71-78.
12. Paolo AM, Bonaminio GA, Gibson C, Partridge T, Kallail K: **Response rate comparisons of e-mail- and mail-distributed student evaluations.** *Teach Learn Med* 2000, **12:**81-84.
13. Torre DM, Simposon D, Bower D, Redlich R, Plma-Sisto P, Lund MR, Sebastian JL: **Learning Activities and Third-Year Medical Student Ratings of High Quality Teaching Across Different Clerkships.** *Med Educ Online* 2006, **11:**32.
14. Dornan T, Arno M, Hadfield J, Scherpbier A, Boshuizen H: **Student evaluation of the clinical 'curriculum in action'.** *Med Educ* 2006, **40:**667-674.
15. Billings-Gagliardi S, Barrett SV, Mazor KM: **Interpreting course evaluation results: insights from thinkaloud interviews with medical students.** *Med Educ* 2004, **38:**1061-1070.
16. Sadoski M, Sanders CW: **Student Course Evaluations: Common Themes across Courses and Years.** *Med Educ Online* 2007, **12:**2.
17. Burton WB, Civitano A, Steiner-Grossman P: **Online versus paper evaluations: differences in both quantitative and qualitative data.** *J Comput High Educ* 2012, **24:**58-69.
18. McOwen KS, Kogan JR, Shea JA: **Elapsed time between teaching and evaluation: does it matter?** *Acad Med* 2008, **83:**S29-32.

19. Cohen-Schotanus J, Schonrock-Adema J, Schmidt HG: **Quality of courses evaluated by 'predictions' rather than opinions: Fewer respondents needed for similar results.** *Med Teach* 2010, **32:**851-856.
20. Woloschuk W, Coderre S, Wright B, McLaughlin K: **What factors affect students' overall ratings of a course?** *Acad Med* 2011, **86:**640-643.
21. Lynoe N, Juth N, Helgesson G: **Case study of a framing effect in course evaluations.** *Med Teach* 2012, **34:**68-70.
22. Purkiss J: **Course evaluation respondents: are 'low-performing retaliators' really over-represented?** *Med Educ* 2012, **46:**513-514.
23. Schonrock-Adema J, Lubarsky S, Chalk C, Steinert Y, Cohen-Schotanus J: **'What would my classmates say?' An international study of the prediction-based method of course evaluation.** *Med Educ* 2013, **47:**453-462.
24. Raupach T, Schiekirka S, Munscher C, Beissbarth T, Himmel W, Burckhardt G, Pukrop T: **Piloting an outcome-based programme evaluation tool in undergraduate medical education.** *GMS Z Med Ausbild* 2012, **29:**Doc44.
25. Schiekirka S, Reinhardt D, Heim S, Fabry G, Pukrop T, Anders S, Raupach T: **Student perceptions of evaluation in undergraduate medical education: A qualitative study from one medical school.** *BMC Med Educ* 2012, **12:**45.
